# Meniscal extrusion: Proposal for a novel qualitative classification

**DOI:** 10.1002/jeo2.70126

**Published:** 2024-12-31

**Authors:** Simone Perelli, Pietro Conte, Nicola Pizza, Rodolfo Morales‐Avalos, Elizaveta Kon, Alberto Grassi, Stefano Zaffagnini, Joan Carles Monllau

**Affiliations:** ^1^ ICATKnee, Institut Català de Traumatologia i Medicina de l'Esport (ICATME)‐Hospital Universitari Dexeus Universitat Autònoma de Barcelona Barcelona Spain; ^2^ Department of Surgery and Morphologic Science, Orthopaedic Surgery Service Universitat Pompeu Fabra, Hospital del Mar Barcelona Spain; ^3^ IRCCS Humanitas Research Hospital Rozzano Milano Italy; ^4^ Department of Biomedical Sciences Humanitas University Pieve Emanuele Milano Italy; ^5^ Department of Physiology, Laboratory of Biomechanics, School of Medicine Universidad Autónoma de Nuevo León Monterrey Mexico; ^6^ Clinica Ortopedica e Traumatologica II IRCCS Istituto Ortopedico Rizzoli Bologna Italy

**Keywords:** degenerative, extrusion, knee, meniscal extrusion, meniscus, osteoarthritis

## Abstract

Meniscal extrusion (ME), defined as the radial displacement of the meniscal body outside the margins of the tibial plateau, has been seen as an independent and relevant predictor of intra‐articular knee degeneration. Nonetheless, available classifications for ME are exclusively quantitative assessments not considering the context in which extrusion is identified. Indeed, ME can be the result of several different conditions spanning from acute tears to chronic degeneration and its definition cannot be only dependent on the numeric calculation of the radial displacement of the meniscal body. Furthermore, growing evidence supports the existence of a paraphysiological ME resulting from joint loading, limb malalignment, anatomical abnormalities of the meniscal attachments to the femur and tibia or a nonpathological finding after meniscal allograft transplantation. It is therefore clear that an exclusively quantitative assessment of ME cannot be sufficient since this condition can develop in such different clinical scenarios. For this reason, a novel qualitative classification for ME is proposed, differentiating between three distinct conditions: a paraphysiological ME, a pathological ME and ME related to degenerative conditions. Furthermore, a comprehensive review of the present literature has been conducted to report the most relevant and updated evidence on the topic highlighting the difference in the clinical management of each different category.

**Level of Evidence:**

Not applicable.

**Abbreviations**
ACL: anterior cruciate ligament
BMI: body mass index
DLM: discoid lateral meniscus
HKAA: hip knee ankle angle
JLCA: joint‐line convergence angle
LMPRT: lateral meniscus posterior root tears
MA: mechanical axis
MAT: meniscal allograft transplantation
ME: meniscal extrusion
MEI: meniscal extrusion index
MME: medial meniscus extrusion
MMPRT: medial meniscus posterior root tears
MPTA: medial proximal tibial angle
MRI: magnetic resonance imaging
MTL: meniscotibial ligament
OA: osteoarthritis
US: ultrasonography

## INTRODUCTION

The medial and the lateral menisci are two crescent‐shaped fibrocartilaginous structures that play a crucial biomechanical role in the knee joint such as axial load distribution, shock absorption as well as joint lubrication, proprioception and stability [35, 78]. Those vital functions can be impaired when lesions occur to either the meniscal tissue itself or to the structures that provide the proper position of the meniscus inside the joint. They are, among others, the anterior and posterior roots and the meniscotibial (MTL), meniscofemoral and meniscofibular ligaments (MFL) [67, 68, 77].

Meniscal extrusion (ME) was originally described by Kenny as the radial displacement of the meniscal tissue, either medially or laterally, outside the margins of the tibial plateau [49] and has recently drawn attention as a relevant factor in the progression of meniscal disfunction. Indeed, when they move from their native position between the tibiofemoral joint and extend in the medial or lateral gutters, the menisci lose their ability to dissipate hoop stress forces thus increasing the contact pressure of the tibiofemoral compartment and consequently enhancing joint degeneration [37, 101]. In the past, some authors also proposed to consider ME as the radial displacement of the meniscus relative to the outer margin of the femoral condyle and that the two measurements, femoral and tibial ME, could differ. Verdonk et al. interestingly reported that when ME is measured from the tibial margin, its value progressively increases going from the posterior to the anterior root while extrusion measured from the femoral margin is greater at the mid body when compared to both anterior and posterior roots [99]. Nonetheless, this femoral‐based reference is nowadays less considered and ME is generally intended as the radial displacement from the tibial plateau.

Several intra‐articular knee pathologies have been correlated with the development of pathological ME such as medial meniscus posterior root tears (MMPRTs) [12, 32], MTLs lesions [38, 53, 68], radial meniscal tears [16, 57, 61] and anterior cruciate ligament (ACL) tears [48]. This phenomenon is also frequently seen after meniscal allograft transplantation (MAT) [1, 45, 66].

Furthermore, ME has been seen as an independent predictor of subsequent cartilage loss, subchondral bone cysts, osteophyte formation and lastly accelerated knee osteoarthritis (OA) [9, 33, 37, 43, 85, 97, 101]. It is worth mentioning that it has also been reported that subchondral bone changes lead to ME [20] thus suggesting that a vicious circle between ME and knee degeneration exists and is still incompletely understood.

ME is generally diagnosed with knee magnetic resonance imaging (MRI) but the fact that this is commonly performed in a nonweightbearing position could lead to an underestimation of the real prevalence and entity of ME. For this reason, both standing MRI [76, 86, 89] and ultrasonography (US) [2, 27, 74] have been proposed and found higher ME values under weight‐bearing conditions [81].

ME is generally classified in a quantitative manner measuring the radial displacement of the meniscal tissue. In 2004, Costa et al. originally proposed an MRI cutoff to categorize between minor (<3 mm) and major (>3 mm) ME [16]. In agreement with this cutoff, a following study found a mean medial meniscal body extrusion of 2.7 mm and a lateral of 1.8 mm in a cohort of healthy volunteers [91] but recent studies proposed different cutoffs for different conditions associated with ME [63]. Two systematic reviews have been recently published on ME quantitative measurement methods: Barreira et al. suggested that the most suitable cut‐offs for pathological ME for both MRI and US seem to fall within >2 and >3 mm [4] whether Farivar et al. highlighted the fact that substantial variation exists in measurement techniques for ME, particularly as it relates to the coronal cross‐sectional reference location, thus posing doubts on the utility of this quantitative evaluation [29]. Indeed, a recent consensus statement from the Meniscus International Study Group, reported that only 53.2% of the members considered the distance in millimetres from the tibial plateau's outer margin as the most reliable measurement technique on imaging [28]. A different calculation method was proposed by Crema et al. based on the percentage of the meniscal body that extruded over the tibial plateau (no extrusion = grade 0; extrusion of <50% of the body = grade 1; >50% of the body = grade 2) [17]. Similarly, Compagnoni et al. recently proposed a further quantitative classification of ME based on the meniscal extrusion index (MEI) calculated by determining the ratio between the extruded portion of the meniscus and the total width of the meniscus [14]. Indeed, they found that there was no relationship between the finite number representing the radial displacement of the meniscus (as proposed by the aforementioned Costa classification) and the percentage of extruded meniscal body given that the meniscal width is highly variable in the population. This supported the adoption of a novel classification rather based on a percentage similarly to the one proposed by Crema et al.: MEI smaller than 20%, MEI between 20 and 40% and MEI > 40%. They further proposed that conditions with a small MEI (<20%) could represent a paraphysiological imaging feature not associated with underlying meniscal lesions whether conditions with MEI > 40% always suggest an underlying lesion that impairs the mechanical function of the meniscus.

Indeed, ME is frequently found in healthy knees of asymptomatic patients and ME quantitatively classified as pathologic could instead represent a paraphysiologic condition.

From the aforementioned evidences, it appears that the mere quantitative classification of ME based on the numeric measure of the meniscal displacement evaluated with MRI is not sufficient and further evaluations should focus on the context in which the ME is identified: the same quantitative value of ME could be found in the context of an acute MMPRT, following a MAT procedure or as a paraphysiological incidental finding thus configuring a different clinical condition.

We therefore propose a novel qualitative classification for ME differentiating between three distinct conditions: a paraphysiological ME, a pathological ME and a ME related to degenerative condition of the meniscus (Table 1). Furthermore, we performed a comprehensive review of the present literature on ME to report the most relevant and updated evidence.

**Table 1:** Proposed qualitative classification of meniscal extrusion and main characteristics of each subtype.

Type of meniscal extrusion	Characteristics	Suggested management
Paraphysiological	Asymptomatic, incidental finding No associated pathological MRI findings Frequently <3 mm No biomechanical effect	No need for surgical correction Conservative management with constant follow up Good prognosis over time
Pathological	Symptomatic, monolateral History of trauma to the index knee Associated lesions: ACL, MTL, roots. Clear extrusion, frequently >3mm Negative biomechanical impact	Needs surgical correction Bad prognosis (OA progression) if not treated Surgical correction of causative lesions leads to good clinical results even if ME persists
Degenerative	Symptomatic, can be bilateral No history of trauma Associated lesions: cartilage wear, BME, osteophytes.	Bad prognosis (OA progression) if not treated No clear therapeutical algorithm Possible options: osteotomy, MAT, replacement surgery

Abbreviations: ACL, anterior cruciate ligament; BME, bone marrow edema; MAT, meniscal allograft transplantation; ME, meniscal extrusion; MRI, magnetic resonance imaging; MTL, meniscotibial ligaments; OA, osteoarthritis.

## PROPOSAL OF A NOVEL QUALITATIVE CLASSIFICATION AND LITERATURE REVIEW

### Paraphysiological ME

**Figure 1:**
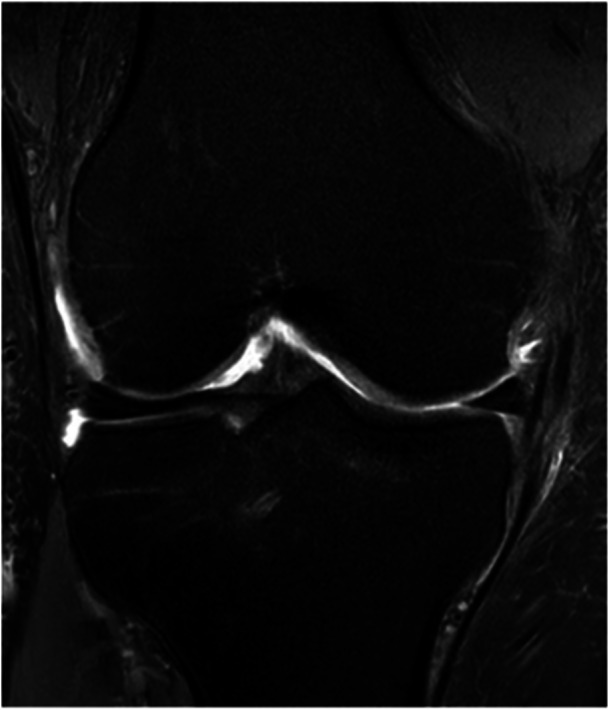
MRI coronal view of a right knee showing a paraphysiological meniscal extrusion. The MRI was performed for patellofemoral pathology and medial meniscal extrusion was detected in an asymptomatic medial compartment. The abnormality of the meniscotibial ligament at this level was detected. MRI, magnetic resonance imaging.

ME is a common finding in healthy knees: in 2006 Puig et al evaluated 100 MRIs of nonarthritic patients and found 68.5% and 18.8% of them having some degree of, respectively, medial and lateral extrusion [80]. Paraphysiological ME (Figure 1), defined as the ME identified in the absence of other knee lesions, has been reported to be more common in the medial meniscus [10], in women [8] and its prevalence increases with age [2]. Several nonpathological conditions can influence the development of a paraphysiological ME and our review of the present literature identified five major distinct conditions: increased axial joint loading, noncommon root insertion, MTL abnormalities, limb alignment and ME found after MAT.

### Joint loading

Axial joint loading is a main factor involved in the radial displacement of the menisci both in healthy and nonhealthy knees. Obesity has been seen as an individual risk factor for medial ME (odds ratio [OR]: 3.04; 1.04–8.93), but not for meniscal lesions (OR 1.15; 0.52–2.54) in a cohort of 644 prospectively followed patients aged 50‐80 years old with a normal medial meniscal status at the baseline evaluation [24]. The impact of weight and body mass index (BMI) on ME was described in several other studies [2, 23] and van der Voet et al. further reported that a 5 kg/m^2^ higher baseline BMI was associated with absolute and relative increase of medial extrusion of 0.2 mm [98]. Liu et al. similarly calculated that each unit higher in BMI was associated with a 13% higher risk of pathologic medial ME [62]. Furthermore, the progression of ME has been seen to diminish with weight loss in two prospective studies on overweight and obese patients [55, 71].

Similarly, extreme joint loading seems to produce a paraphysiological ME also in nonobese healthy patients: Diermeier et al. performed both MRI and US on 18 healthy athletes before, at the conclusion and 2 weeks after a mountain ultramarathon. In this cohort of asymptomatic patients with no history of knee surgery, ME increased significatively during the race (with mean values above the aforementioned 3 mm cut off) and then showed a complete recovery 2 weeks after the end of the race [19]. Similarly, Ishii et al. recently evaluated on 30 healthy patients the effects of cumulative stress during walking and jogging in different environments and found that temporary extrusion of the meniscus (evaluated with US at baseline, post effort and 1 day after the activity) occurred after uphill/downhill tasks and that this reaction was observed only in the medial meniscus [44]. Lastly, Rennie and Finley compared asymptomatic young rugby and soccer players to a control group of nonathletes and found higher frequencies of ME in athletes (48% vs 30%) when the 3 mm cut‐off value was adopted further supporting the idea that asymptomatic ME is a common finding in healthy joints subjected to frequent axial loading [82].

### Noncommon anterior root insertion

In 1998 Berlet and Fowler performed a cadaveric study on 48 knees and proposed a classification for different insertions of the anterior horn of the medial meniscus [6]. They found that type III (on the anterior slope of the tibial plateau) and IV (no firm insertion on the plateau) were found respectively in 15% and 3% of the specimens and reported that those noncommon insertions were thought to be unable to resist peripheral extrusion during meniscus loading. Accordingly, in the aforementioned study of Puig et al., on 100 healthy knees MRIs, medial ME was significatively correlated to an anterior variant of the medial meniscus insertion on the tibial plate classified with a simplified version of the classification proposed by Berlet and Fowler [80].

### MTL abnormalities

ME can be found in knees with intact menisci and can be a result of nontraumatic abnormalities in the structure and integrity of the MTLs. Krych et al. reviewed 3244 MRIs of patients with ME and found that 20 of those (0.62%) had an isolated ME (meaning extrusion in the absence of any meniscal lesion) and minimal concomitant knee pathology with 45% of those patients presenting >3 mm of ME [53]. 13/20 of the evaluated patients with isolated ME (100% of those with >3 mm ME) had MTL abnormalities defined as ligaments that demonstrated increased signal and/or an attenuated appearance. Thus, it was suggested that in cases of ME > 3 mm, intact meniscus and minimal knee pathology, an abnormality to the MTLs should always be suspected. The clinical implication of this finding was recently addressed by the same group performing serial MRIs in patients with medial meniscus extrusion (MME) that later presented an MMPRT: [54] 26 patients with a first MRI reporting MME, intact meniscus and no history of knee trauma and a following MRI highlighting a MMPRT were included. Interestingly, it was reported that, at the first MRI, all the included patients demonstrated clear medial ME and MTL disruption before the subsequent development of MMPRTs reversing the common paradigm of ME being considered a consequence of MMPRT. It is worth mentioning that the mean age of those 26 patients at the time of the first MRI was 55.7 ± 9.9 years thus raising the suspect of possible degenerative ME, later discussed in the manuscript, but the authors did not mention any sign of degeneration at in the index compartment at the initial MRI.

Accordingly, Nishino et al. compared MRI of symptomatic and asymptomatic patients (mean age 15.8 ± 9.2 years) with discoid lateral meniscus (DLM) and reported that MTL loosening was independently associated with ME and was significantly more frequent in the symptomatic DLM group [73]. Ekşili et al. reported that the damage of both MTL and meniscofemoral ligament was significantly associated with MME in a cohort of patients without any acute injury and any disease that could disrupt the meniscal anatomy detected on knee MRI [23].

Nonetheless, the MTLs are not the only relevant structure attaching to the meniscal body preventing ME. Masferrer‐Pino et al. conducted a cadaveric study to precisely identify the peripheral attachments of the lateral meniscal body acting as restrictors of lateral ME [67]. The study described a consistent anatomic pattern between the fibres of the lateral MTL, the popliteofibular ligament and popliteomeniscal ligament forming an interconnected complex with the proposed name of menisco‐tibio‐popliteus‐fibular complex. The same complex was subsequently described in vivo by the use of knee MRI [70].

### Limb alignment

The impact of knee alignment on the onset of ME is still controversial [36]. Goto et al. tried to determine the relationship between alignment parameters and MME retrospectively reviewing 190 knee MRIs of patients with intra‐articular knee disorders [39]. All the considered alignment factors (hip knee ankle angle: HKAA, percentage of mechanical axis, medial proximal tibial angle: MPTA and joint‐line convergence angle: JLCA) significantly correlated with MME suggesting that varus alignment factors are related to ME. Interestingly, the separate analysis for major (>3 mm) and minor (<3 mm) ME found that the correlation was only present in cases with ME > 3 mm and that minor (paraphysiological) ME was not correlated with alignment parameters.

With the same intent of evaluating whether an alteration of the lower limb axis is associated with ME, Erquicia et al. compared 94 patients with either mild varus or valgus knee deformities (HKAA 174°–186°) and no relevant intra‐articular knee pathology [25]. They found that only 17.3% of the medial meniscus and 26% of the lateral meniscus presented major extrusion (>3 mm), respectively, in varus and valgus‐aligned knees. Furthermore, no correlation was found between minor or major and medial or lateral meniscus extrusion and the kind and entity of knee alignment. Conversely, van der Voet et al. reported that the presence of varus malalignment (defined by HKAA < 178°) was associated with an overall increase in medial ME of 0.6 mm in a population of overweight women whether a valgus malalignment (defined by HKAA > 182°) produced no effect on lateral ME [98]. On the other hand, Crema et al. reported that both varus (HKAA < 178°) and valgus (HKAA > 182°) malalignment were associated, respectively, with medial (OR: 1.3; 1.1–1.7) and lateral (OR: 2.2; 1.5–3.2) MEs [17].

Lastly, Kozaki et al. suggested that the combined relationship between ME and alignment measures should be considered when trying to determine the clinical relevance of ME: with a finite element analysis, they determined that the stress loading on the medial compartment produced by ME is enhanced with a varus alignment of the JLCA but is not relevant when JLCA has a valgus alignment even in the presence of >3 mm of ME [52].

### Post MAT ME

**Figure 2:**
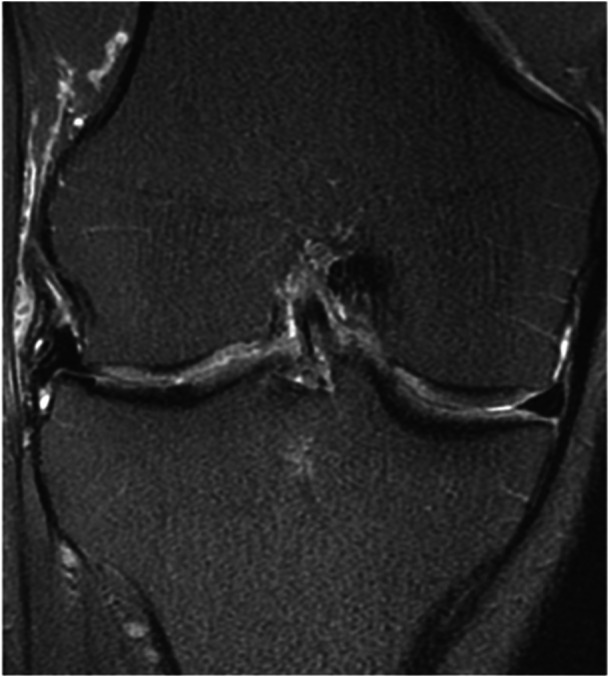
MRI coronal view of a right knee at 5 years follow‐up post lateral meniscal transplantation. An almost complete meniscal body extrusion can be observed. Despite this radiological finding the patient is completely asymptomatic. MRI, magnetic resonance imaging.

Nowadays, minor ME found in the index compartment after MAT (Figure 2) is frequently considered a paraphysiological condition with no relevant clinical consequences [96]. Indeed, Song et al. retrospectively reviewed 97 lateral MAT at minimum 5 years follow up dividing the cohort based on the entity of ME (<3, 3–5 and >5 mm) [88]. They reported that the variation in joint space width, and therefore the OA progression, was different between ME <3 and >3 mm but not between 3–5 and >5 mm with an acceptable amount of graft extrusion found to be 3.74 mm (sensitivity, 81.8%; specificity, 77.8%). Nonetheless, no significant difference in clinical outcomes was found according to the amount of graft extrusion. In a previous, short‐term, follow up of the same cohort they had further demonstrated that meniscal allograft extrusion had no influence in postoperative changes in meniscus width, thickness, and relative intrameniscal signal intensity [87]. The same authors reported similar results (a difference in the variation of joint space width but no difference in clinical score between <3 and >3 mm ME) also for medial MAT at a mean of 12 years of follow up [59]. Similarly, Kim et al. reported that, in their cohort of 30 MATs, extrusion (here reported as the percentage of the meniscal body extruding outside the tibial plateau) had a mean value of 41%, remained stable between the 3 and 12 months follow up, and had no correlation with early post‐operative clinical outcomes [50]. Based on these evidences, it is clear that mean extrusion after MAT is definitely higher than that of the native meniscus and that therefore extrusion values considered to be pathological for the native meniscus (e.g., 3–4 mm) are to be considered paraphysiological after MAT procedures [5, 88, 96]. Furthermore, minor extrusion after MAT seems to be exclusively correlated with radiological progression whether clinical results remain good regardless of the extrusion rate [50, 59, 87, 88].

The choice between different surgical fixation techniques for MAT and their impact on graft extrusion control remains controversial. Three literature reviews performing a comparison in ME with bone‐bridge and soft‐tissue MAT fixation were published in the last 2 years: Worhacz and Carter conducted a narrative review and stated that bone‐bridge techniques seem to provide less extrusion for both medial and lateral MATs [103] whether Leite et al. performed a systematic review of 24 studies (328 medial MATs) and concluded that allograft extrusion appears equivalent for both bone plug and soft‐tissue fixation techniques [60]. The most recent systematic review on the topic was performed by Beel et al. and reported that graft extrusion was a common finding after medial and lateral MAT, independent of the root fixation technique with bony fixation resulting in lower or equal extrusion when compared to soft‐tissue fixation: mean absolute extrusion values of 3.2 for soft and 3.36 mm for bony fixations and 3.72 for soft and 2.78 for bony fixations for medial and lateral MATs, respectively, were found [5]. Comparable results were also found in a retrospective analysis of 112 lateral MATs made by Bhattacharyya et al. [7]. Furthermore, a meta‐analysis of 38 studies (1637 MATs) from Jaiuregui et al. reported no difference between soft tissue suture and bone fixation for MAT root fixation regarding clinical outcomes, failures and ME [46].

It was also suggested that the addition of a capsulodesis significantly reduced ME in lateral MAT fixed with a suture‐only technique [66] and it was recently reported that when a capsulodesis is applied, ME remain stable at a mid‐term follow up with no difference between 2 and 7 years of follow‐up [69].

Even if the clinical impact of minor graft extrusion after MAT procedures seems to be limited, several surgical techniques have been proposed to reduce ME after MAT with the aim of better replicate the native meniscus anatomy and reduce the radiological impact of extrusion: different capsulodesis techniques [58, 66, 84], MTL reconstruction [15], a third bony tunnel [95] and the tenodesis of the intermeniscal ligament [42, 51]. All of those were shown to reduce meniscal allograft extrusion but their impact on the osteoarthritic progression is yet to be proven.

## PATHOLOGICAL ME

**Figure 3:**
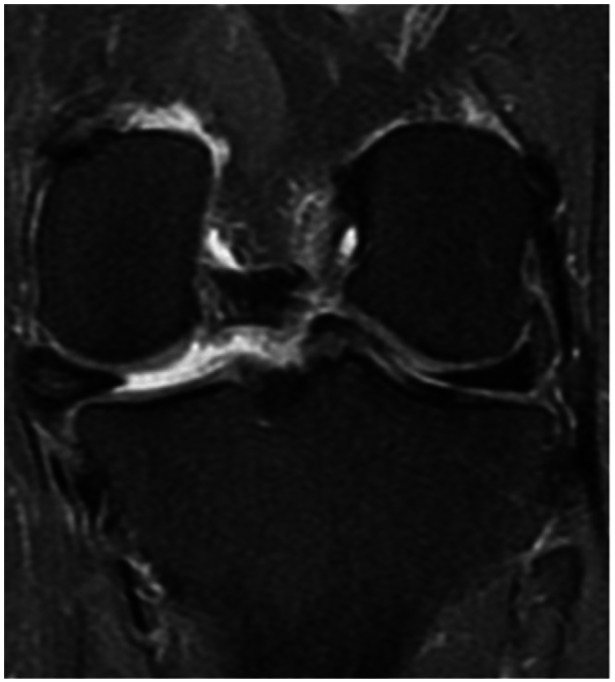
MRI coronal view of a left knee. Posterior medial root tear lesion associated with meniscal extrusion. MRI, magnetic resonance imaging.

It has been thoroughly demonstrated that several types of meniscal lesions can produce ME through the disruption of meniscal function and that the surgical treatment of those lesions is essential to reduce the otherwise inevitable onset of knee OA [17, 65, 75, 92]. MMPRTs, radial tears and lesions of the menisco‐tibial ligaments seem to be the most frequent and clinically relevant [61, 62, 64, 65, 105] (Figure 3).

### Meniscal root tears

MMPRT is considered the most frequent and relevant pathological meniscal condition leading to ME [32, 61, 72, 105]. Clinical studies and meta‐analysis regarding surgical MMPRT repair, although supporting its superiority over arthroscopic partial meniscetomy, seem to report only a limited ability to successfully reduce ME in the setting of transtibial pull‐out MMPRT repair [13, 26, 31, 93]. In a recent systematic review and meta‐analysis of both biomechanical and clinical studies, Perry et al. found that surgical repair of MMPRT is not able to significantly correct ME but is nonetheless capable of improving both clinical outcomes and joint biomechanics and lead to a limited OA progression [79].

Recently the focus has been moved on the development of additional peripheric procedures to limit ME following MMPRT [56]. Amano et al. demonstrated that the augmentation of a nonanatomical repair of MMPRT using three peripheric knotless anchors may be associated with less ME and better compressive load distribution in a porcine model [3]. Similarly, Daney et al. demonstrated that the addition of a centralization suture to an anatomical transtibial pullout MMPRT repair was able to reduce pathologic ME and restore joint contact mechanics in an human cadaveric model [18]. Boksh et al. recently collected the evidences on the effect of centralization procedures in the setting of meniscal surgery in a systematic review and meta‐analysis and reported that centralization procedures significantly improves clinical scores (KOOS and Lysholm scores) and significantly reduces ME at mean 17 months postsurgery [11].

Similar results have been found also with lateral meniscus posterior root tears (LMPRT), frequently found during ACL reconstruction procedures: [34] the surgical repair of LMPRT seems to restore joint mechanics and improve clinical outcomes but seem to be ineffective in reducing ME [79, 106].

### Radial meniscal tears

**Figure 4:**
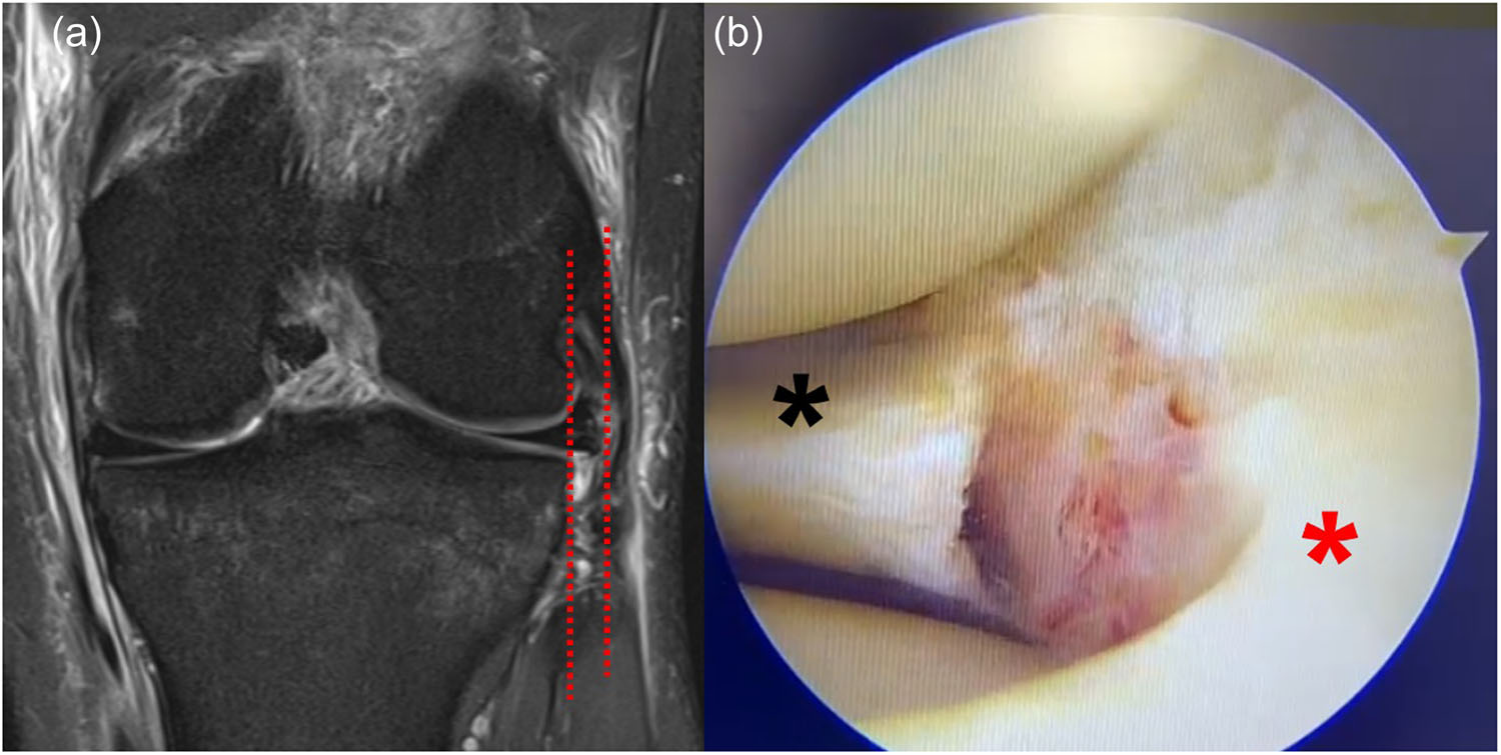
(a) MRI coronal view of a left knee. The red‐dotted lines represent the amount of meniscal extrusion. (b) intraoperative arthroscopic view of the radial lateral meniscal lesion, the red and black asterisk pointed out the two meniscal segments. MRI, magnetic resonance imaging.

ME appears to be greater when a radial component is present in a meniscal tear [57, 105] with ORs reaching as high as 4.34 for such tears compared to horizontal tears [62]. Nonetheless, evidence on the impact of meniscal repair of radial tears in ME reduction is still limited: Yeh et al. reported no identifiable progression and Winkler et al. a reduction in ME following the repair of lateral meniscus radial tears with all‐inside arthroscopic procedures [102, 104]. Probably the increase of ME when a radial meniscal tear is present could be due to the involvement of the menisco‐tibial ligament at the level of the meniscal tear (Figure 4).

### MTL rupture

**Figure 5:**
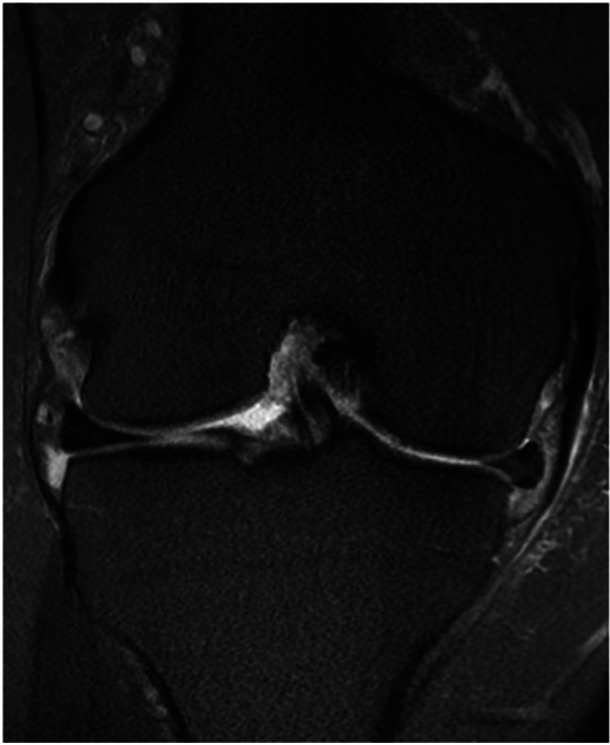
MRI coronal view of a right knee. Patient with valgus instability due to a medial collateral ligament grade 3 lesion 6 months before. In the MRI are visible both the scarred medial collateral ligament at the femoral insertion and the lesion of the meniscotibial ligament with concomitant extrusion. MRI, magnetic resonance imaging.

Acute MTL lesions are thought to be strongly associated with ME (Figure 5). A systematic review and meta‐analysis from Gilat et al. found six studies evaluating the role of either acute MTL rupture or induced MTL lesions [38]. MTL injuries were reported to lead to approximately 3 mm of MM extrusion, while their repair seemed to decrease extrusion by 2 mm. Two recent cadaveric studies were not included in this systematic review: Doan et al. demonstrated that an MTL tear resulted in significant ME without a detectable difference in medial compartment pressures [21] whether Farivar et al. reported that an isolated MTL tear is able to cause 2–2.99 mm of ME [30]. Morales‐Avalos et al. recently showed that in a porcine model, the lateral MTL and the MFL) participate as restrictors of the radial mobility of the lateral meniscus during loading. Their injury caused a significant increase in lateral ME, and the centralization or capsulodesis procedures were able to reduce extrusion [68].

## DEGENERATIVE ME

ME is also clearly associated with aging [2, 23, 28, 37, 43, 47] but whether ME is to be considered either a cause or a consequence of articular degeneration is still of open debate. Indeed, different conditions such as ME, meniscal lesions, chondral lesions and ACL lesions clearly contribute to the development of knee OA [105] but the relative impact of each of them is still unclear and object of investigations. Furthermore, those conditions are found to be associated with OA but it is frequently challenging to clearly demonstrate that those are actually causative of OA progression and not an epiphenomena of it. Indeed, several of those degenerative processes, such as osteophyte formation and ME, appear together in longitudinal studies and it is therefore difficult to determine if one is causing the other or if both are consequence of a third, broader, process [40, 41].

**Figure 6:**
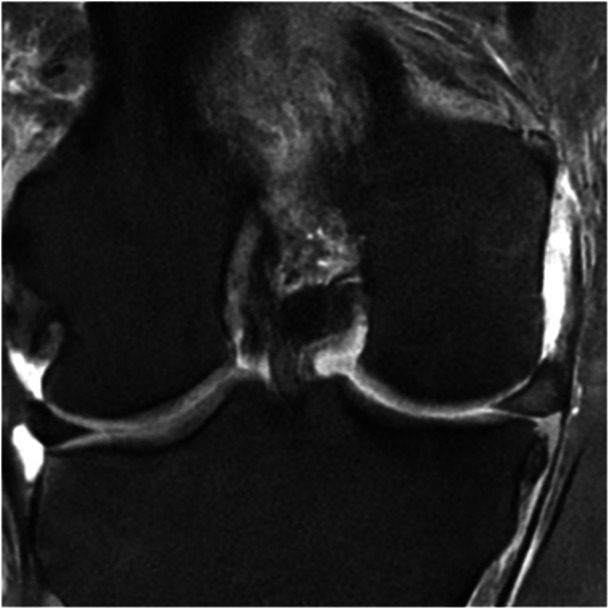
MRI coronal view of a right knee. Medial ME as well as signs of medial meniscal degeneration can be observed. A mild reduction of tibial chondral thickness is visible without subchondral changes nor osteophytes. ME, meniscal extrusion; MRI, magnetic resonance imaging.

Indeed, ME can develop in a degenerative, nontraumatic, setting when the original meniscal fibrocartilagineous structure is altered by progressive and chronic modifications (Figure 6). The resultant ME cause a reduction of the meniscal ability to dissipate hoop stress thus increasing contact pressure and leading to the development of chondral lesions and bone marrow lesions that produce further stress and displacement of the meniscal tissue [20, 83, 90]. This triggers a vicious circle in which the failure of several protective structures and the alteration of joint homoeostasis facilitate the degeneration of the entire joint thus ending in the progression of osteoarthritis.

Nonetheless, baseline ME has been seen as an independent risk factor for osteoarthritis progression in several longitudinal studies monitoring the progressive onset of knee OA [3, 20, 22, 63, 85, 94, 100]. Thus, even if this relationship seems to be bidirectional, ME, especially if traumatic, is more frequently one of the causes of joint degeneration rather than its consequence but degenerative ME deserves a greater consideration.

## DISCUSSION

The purpose of this classification was to understand the pathophysiology underlying the phenomenon of ME. This phenomenon, frequently observable radiologically, is not always pathological. Furthermore, in some patients in whom we can define the extrusion as quantitively pathological, surgical treatment is not always mandatory. On the contrary, in patients who present a paraphysiological extrusion but are symptomatic for the specific characteristics of the patient, a surgical solution could be considered. Understanding the pathophysiological mechanism underlying extrusion can allow us to understand when the surgical treatment is the most appropriate, even if precise indications regarding the best surgical treatment are not yet available in the literature. Defining a specific treatment protocol for each type of extrusion is beyond the scope of this review, especially considering the absence of precisely defined protocols in the literature. The general recommendation of the authors of this review based on the proposed classification is to consider paraphysiological extrusion as a phenomenon not to be treated surgically but to be monitored over time as it is not necessarily biomechanically unfavourable. If it was symptomatic, the associated risk factors should be corrected first (obesity, malalignment, etc.). On the other hand, pathological extrusion it is often traumatic, even in those cases in which the lesion is developed in the context of a degenerated meniscal tissue (e.g., posteromedial root tear). Therefore, it should be considered for surgical repair taking in account both the acute symptoms and the possible long‐term consequences. Degenerative extrusion is the most complex to understand and manage, since it often appears in cases of osteoarthritis where surgical treatment of the meniscal problem alone may be incomplete. In these patients, each case must be considered individually. Future clinical studies are necessary to properly indicate which surgical treatments are most suitable for each type of extrusion and the related long‐term results.

## CONCLUSIONS

ME has become a relevant theme in the field of meniscal and knee degeneration research but certainties and agreement on its clinical management are still lacking. Specifically, quantitative imaging measures appear insufficient in both diagnosing ME and determine when and if this condition needs to be surgically addressed. In this manuscript, we propose a novel qualitative classification of ME describing the peculiarities of paraphysiologic, degenerative and pathologic ME through a review of the most relevant and up‐to‐date available literature. Further research will need to separately address those different conditions to better understand their different clinical impacts and produce validated and commonly agreed management protocols.

## AUTHOR CONTRIBUTIONS

Material preparation, data collection and analysis of the available literature were performed by Pietro Conte, Nicola Pizza and Rodolfo Morales‐Avalos with the constant supervision of Simone Perelli and Alberto Grassi. The first draft of the manuscript was written by Pietro Conte, Nicola Pizza and Simone Perelli. Then, Stefano Zaffagnini, Elizaveta Kon and Joan Carles Monllau commented on previous versions of the manuscript and gave their expert opinion to refine the manuscript and the proposed classification. All authors contributed to the study's conception and design. Finally, all authors read and approved the final manuscript.

## CONFLICT OF INTEREST STATEMENT

The authors declare no conflicts of interest.

## ETHICS STATEMENT

An ethics approval was not needed for this study and article.

## Data Availability

Data sharing is not applicable to this article as no clinical data sets were generated or analysed during the current study.
